# Reducing leakage current and dielectric losses of electroactive polymers through electro-annealing for high-voltage actuation

**DOI:** 10.1039/c9ra01469a

**Published:** 2019-04-26

**Authors:** Francesco Pedroli, Alessio Marrani, Minh-Quyen Le, Olivier Sanseau, Pierre-Jean Cottinet, Jean-Fabien Capsal

**Affiliations:** Univ Lyon, INSA-Lyon, LGEF, EA682 F-69621 Villeurbanne France jean-fabien.capsal@insa-lyon.fr; Solvay Specialty Polymers viale Lombardia 20, 20021 Bollate Italy; P2D, CNRS/Rhodia-Solvay, UMR 5268 85 avenue des Frères Perret, F-69192 Saint Fons France

## Abstract

Electroactive polymers (EAPs) such as P(VDF-TrFE-CTFE) are very promising in the field of flexible sensors and actuators. Their advantages in smart electrical devices are due to their low cost, elastic properties, low density, and ability to be manufactured into various shapes and thicknesses. In earlier years, terpolymer P(VDF-TrFE-CTFE) attracted a lot of research due to its relaxor-ferroelectric property that exhibits high electrostriction phenomena. While widely used in flexible actuation, this class of material is still limited by the high electric fields required (≥30 V μm^−1^) to achieve sufficient strain levels (>2%). This inevitably leads to high levels of leakage current and thus a short lifetime. This paper proposes a new approach based on electro-annealing thermal treatment for a pure terpolymer P(VDF-TrFE-CTFE) matrix in order to limit the conduction mechanisms. This in turn reduces the dielectric losses at a high level of electric fields. The experimental results demonstrate that a huge decrease in leakage current of 80% is achieved for a wide range of electric fields (*i.e.* up to 90 V μm^−1^) with a 4-fold extension in time-to-breakdown at high voltage excitations of 40 V μm^−1^.

## Abstract 2

The electro-annealed polymer, the E-TH sample, shows a reduction in leakage current of 80% for very high electric fields.

## Graphical abstract

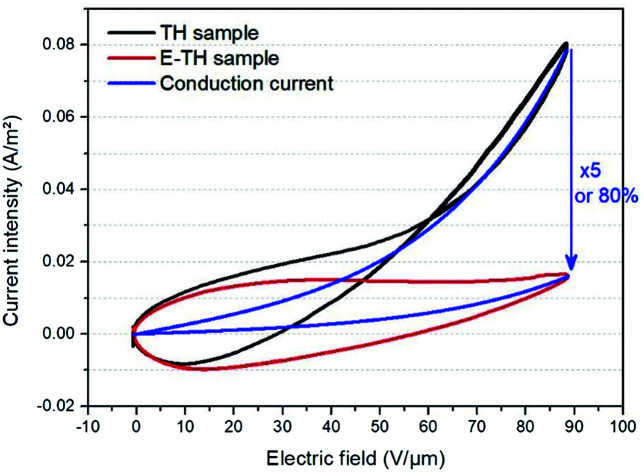

## 1. Introduction

Electroactive polymers (EAPs) are attractive candidates for next-generation micro-electromechanical systems (MEMs) and smart actuators due to their easy processability with large and complex shapes, light weight,^1,2^ fast electromechanical response, and low mechanical and acoustic impedance.^3,4^ The peculiarity characterizing this class of materials is their ability to change shape upon an external stimulus such as electric voltage, thermal variation, and/or light exposure.

EAPs can be classified into two main categories, *i.e.* ionic and dielectric. For both electro-active polymer types, actuation is driven by an applied electric field, but the material deformation is steered by very different physical mechanisms. The working principle of the ionic EAPs is based on ionic exchange between an electrolyte and a polymer matrix upon the appliance of the electric field; in the case of dielectrics, polymer deformation is driven by the electrostatic force between the two electrodes generated by the external electric field.^5^ Here we focus only on this second category of polymers because they have wider versatility and a faster response to electrical stimuli—this makes them more promising in the development of active actuators. Unlike ionic polymers, dielectrics do not need an environment rich in ionic species to operate. Thus, they can be implemented in several kinds of atmospheres.^6^

Of the existing dielectric EAPs (silicones, acrylates, polyurethanes and PVDF-based polymers), the most performing one in terms of electromechanical conversion is fluorinated electrostrictive terpolymer P(VDF-TrFE-CTFE).^7–10^ The small value of Young's modulus of silicones and acrylates such as polydimethylsiloxane (PDMS) and polyurethane (PU) allows these polymers to reach very large strains.^11^ At the same time, it severely limits the electro-to-mechanical energy conversion.^12,13^ The semicrystalline terpolymer P(VDF-TrFE-CTFE) exhibits extremely large elastic energy density.^14,15^ Its peculiar semicrystalline morphology leads to strongly enhanced polarization levels thanks to the presence of cooperative nanopolar regions in crystalline domains.^16,17^

Despite the augmented electrostrictive performances of fluorinated dielectric polymers, high electrical fields are still required to attain satisfactory strain levels.^1^ The driving voltage is proportional to dielectric thickness, and electroactive polymer films remain in the range of tens of micrometers. They did not go beyond those levels of driving voltage that can represent a strict limitation to wide industrial and commercial applications.^3,18^ Thin polymer films with high quality and homogeneity are now achievable and scalable (even at very low thicknesses on the order of micrometers).^19–23^ However, the use of high electric fields with low-glass-transition polymers inevitably results in a high level of leakage current.^24^

The control of dielectric losses and their limits in terms of an exponential increase at operating voltage is fundamental to the development of high performing and long-life polymeric actuators.^25^ High levels of leakage current also increase energy dissipation^26^ and underlies self-heating.^27,28^ This promotes material degradation processes^29–31^ and leads to a dramatic drop in material dielectric strength or electrical breakdown. This severely reduced electrical breakdown does not allow EAPs to fully assess their potential for target applications such as energy storage or actuation.

The main objective of this work involves developing long-life polymeric actuators by perfectly controlling the dielectric mechanisms governing ionic conduction in pure P(VDF-TrFE-CTFE) terpolymers. To enhance its intrinsic electrical properties, we propose here a new electro-thermal annealing process for semicrystalline terpolymer. A 2-fold reduced ionic conductivity at low voltage input has been recorded at 0.1 Hz along with a 5-fold reduced leakage current at high voltage input (up to 90 V μm^−1^). Structural analysis, dielectric spectroscopy, and electrical characterization can identify the physical phenomena behind this decrease in conductivity. In addition, aging tests and electromechanical measurements can better assess the material performance—especially in terms of smart actuator design.

## 2. Materials and process

### 2.1 Sample fabrication

The semicrystalline P(VDF-TrFE-CTFE) terpolymer used in this work was synthesized *via* a micro-emulsion polymerization process^32^ and provided by Solvay Specialty Polymers Italy S.p.a. Terpolymer films can be realized *via* solution-casting or doctor-blading. The selection of polymer grade—including monomeric composition, molecular weight—as well as optimization of solvent, filtration, drying, and annealing steps have been described in our previous work.^33^ An optimized average molecular mass of terpolymer was found with superior homogeneity and good electrical and mechanical responses.

Films were prepared by casting a dissolution of 25 %wt. terpolymer powder dissolved in 2-butanone (also known as methyl ethyl ketone, or MEK). Before casting, the dissolution was filtered *via* a pressure column system equipped with polytetrafluoroethylene (PTFE) membrane filters with sub-micron pore dimensions. The polymeric dissolution was then cast on tempered glass plates and dried at room temperature. Prior to thermal treatment, the film was peeled away from the glass plate and laid down on PTFE foils to not induce any internal residual stress due to the different thermal expansion coefficients of terpolymer and tempered glass. To ensure complete solvent evaporation even in the case of thick film deposition (*i.e.* 90 μm), the prepared film underwent a first low-temperature thermal treatment 60 °C in a convection oven. We note that such a temperature did not lead to modifications in crystal morphology nor did it promote crystal growth. Finally, circular gold electrodes 20 mm in diameter and 17 nm-thick were coated on both side of the terpolymer film to obtaining a metal/polymer/metal capacitor-like architecture. Deposition used gold sputtering (Cressington 208 HR).

### 2.2 Thermal annealing and electro-thermal annealing

**Fig. 1:**
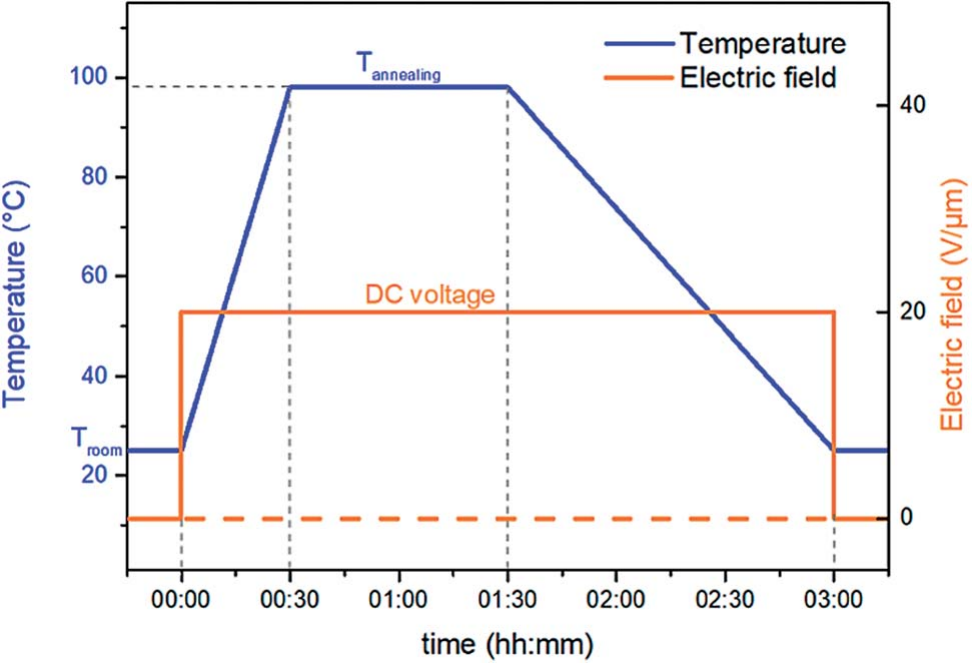
Temperature and voltage profile for the standard thermal and the novel electro-thermal annealing treatments.

Thermal treatment at higher temperature is called annealing and it greatly promotes crystal growth and crystal size homogeneity.^34^ Thus, to enhance the ferro-relaxor behavior of semicrystalline P(VDF-TrFE-CTFE), a new thermal annealing concept was investigated here. This treatment was performed in a convection oven with optimal annealing temperature determined *via* differential scanning calorimetry (DSC) as the onset of melting peak (98 °C). The novel idea proposed here combines thermal annealing with an electrical input to create so-called electro-thermal annealing. The treatment used electrode-fitted samples with a constant DC voltage corresponding to an electric field of 20 V μm^−1^. This was applied throughout the annealing process. The sample was heated at 98 °C for about 1 h and then cooled down to room temperature while holding the DC voltage power constant. Fig. 1 illustrates the temperature and voltage profile for both thermal and electro-thermal annealing treatments.

Two samples were prepared based on the two different thermal processes: samples prepared with the standard annealing (“TH”) and samples prepared with novel electro-thermal annealing (“E-TH”).

## 3. Characterization methods

### 3.1 Morphological characterization

Three different analytical techniques were used based on the magnification scale. DSC characterized the crystalline phase in terms of crystal content, average crystallite size, and crystallite size distribution. For deeper study of the annealing effects of both TH and E-TH samples on polymer morphology, structural characterizations at smaller scales were performed like X-ray diffraction (XRD) and infrared spectroscopy (IR). The XRD checked the crystal-phase quality by comparing positions and width at half maximum (FWHM) of the characteristic peaks to confirm a possible change in crystalline structure. Complementarily to XRD, IR verified the probable chemical modifications due to charge injection through the film surfaces under high electric field^35–37^ together with high temperature treatment, *i.e.* during the electro-thermal annealing. Indeed, the IR spectral peak positions were related to the vibrational resonance frequency of chemical bonds in a certain chemical surrounding. In the case of polymer degradation, structural variation such as the formation of double bonds or macromolecule scissoring could be detectable *via* modifications in the resonance spectra.^35,38^

DSC used a SETARAM DSC131 EVO calorimeter. The thermal characterization determined the potential effects on crystal morphology induced by the two different annealing treatments mentioned previously. The first series of post-annealed samples was heated from room temperature to 140 °C at 10 °C min^−1^. Subsequently, a second series was analyzed with a faster heating rate of 20 °C min^−1^ to ensure no modifications to the crystal morphology during the heating ramp. The thermograph for the second melting of the terpolymer sample showed that the optimal annealing temperature was identified as the temperature onset at melting peak.^39^ Finally, the crystallinity degree (or *χ*_c_) of the post-annealed samples was calculated from integration of the melting peak divided by the enthalpy fusion of a hypothetic 100% crystal of terpolymer (*i.e.* 42 J g^−1^).^40^

The XRD was performed *via* an X'Pert Pro MPD Panalytical diffractometer using Cu-Kα radiation (*λ* = 1.5406 Å) of 45 kV and an electrical input of 40 mA in tandem with an incident-beam monochromator (Inc. beam Johansson 1xGe111 Cu/Co) and a X'Celerator detector. The diffraction patterns were recorded over an angular range of 10–30° (2*θ*) where the characteristic peak relative to P(VDF-TrFE-CTFE) crystalline phase was localized.^40^ A step length of the angular (2*θ*) equaled 0.017° with a counting time of 120 s per step. The extraction of the peak positions for indexing was performed *via* the X'Pert High Score.

The IR spectroscopy used “attenuated total reflectance” (ATR) because the spectra recorded *via* transmission of IR rays across the sample thickness were saturated. Measurement were achieved *via* a Spectro IR Alpha analyzer (Bruker) equipped with a ATR Diamant tip from 4000–400 cm^−1^ with a resolution of 4 cm^−1^ and 32 scans.

### 3.2 Dielectric losses

Broadband dielectric spectroscopy (or BDS) was obtained using SOLARTRON 1260 impedance-analyzer. The dielectric spectra were acquired under AC electric voltage of 1 V_peak–peak_ and frequency range of 10^−1^ to 10^6^ Hz at room temperature. BDS is a helpful tool to evaluate ionic conductivity and directly measure the dielectric losses of EAPs.^41^

### 3.3 Aging test

The aging tests were performed over 22 hours. The samples were prepared with two different processing fabrication. Unipolar sinusoidal electric fields of 40 V μm^−1^ and 100 mHz frequency were applied along the thickness of the terpolymer films; the resulting current was recorded in real-time. In addition, similar to all other electrical characterization, samples were soaked in dielectric silicon oil to offer a double advantage to the measurement approach. First, it prevents both electrical discharges in air and polymer film contouring by superficial currents. This can generate electrical shortcuts interrupting the power supply. Second, the thermal insulating properties of silicon oil hinder the thermal exchange from the sample surfaces and enhance the self-heating processes that drive leakage currents.

### 3.4 Electromechanical characterization

**Fig. 2:**
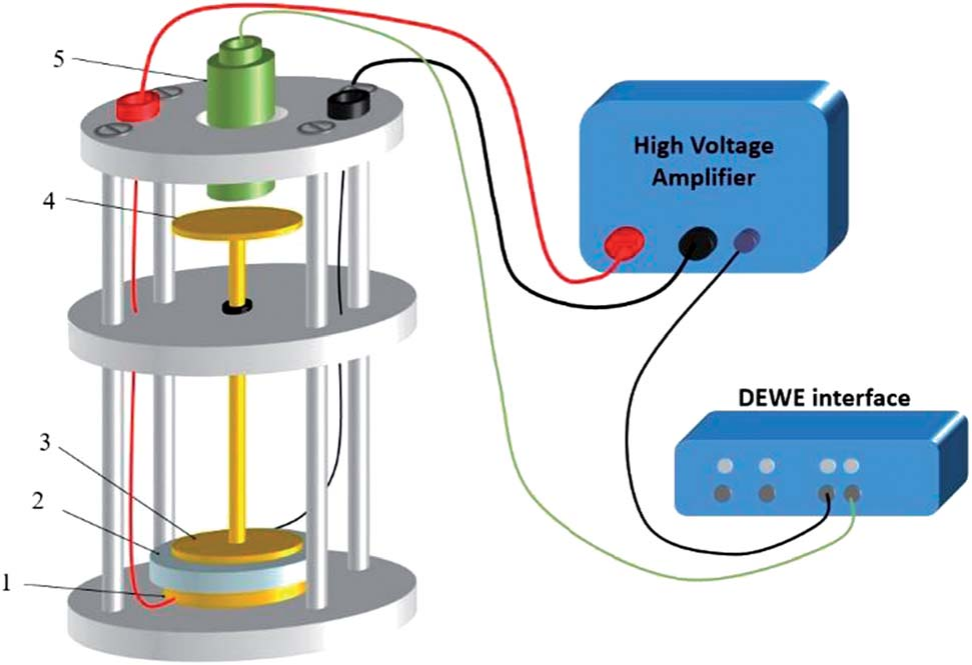
Experimental setup for the electromechanical characterization of EAPs: (1) input electrode, (2) EAP sample, (3) ground electrode or moving electrode, (4) moving disk, (5) capacitive displacement sensor.

A dedicated setup for measuring the electromechanical performances of terpolymer samples is shown in Fig. 2. Samples were placed between the input electrode (1) and the ground electrode (3). To avoid measurements of parasitic deformations, the clamping electrodes should have the same diameter of the gold electrode (*i.e.*, around 20 mm). The input voltage was generated by a waveform generator (Agilent 33220A) that was then amplified by a voltage amplifier (Trek 20/20C). Electromechanical actuation of terpolymer sample was performed under two different configurations of excitation, *i.e.* AC bipolar sinusoidal signal of 50 mHz frequency, a peak-to-peak amplitude corresponding to 20 V μm^−1^, and unipolar signal of 100 mHz with an amplitude up to 90 V μm^−1^.

We emphasize that this setup can monitor the resulting current and the actuation displacement at the same time. The current amplification used a Stanford SR-570 amplifier connected to the moving electrode; the displacement was achieved *via* a high-precision capacitive sensor (FOGALE MC 940). The measured displacement referred to the thickness variation Δ*d*^E^ driven by a input voltage excitation (longitudinal displacement) because it parallels the electric field direction (*i.e.* the 33-direction).^42^ Accordingly, the longitudinal strain *S*_33_ was given by:1
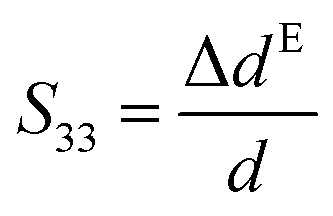


Finally, all signals including displacement, voltage, and current were simultaneously recorded in real-time using DEWE software (Sirius 8XSGT). Post-data treatment was performed thanks to Origin software.

**Fig. 3:**
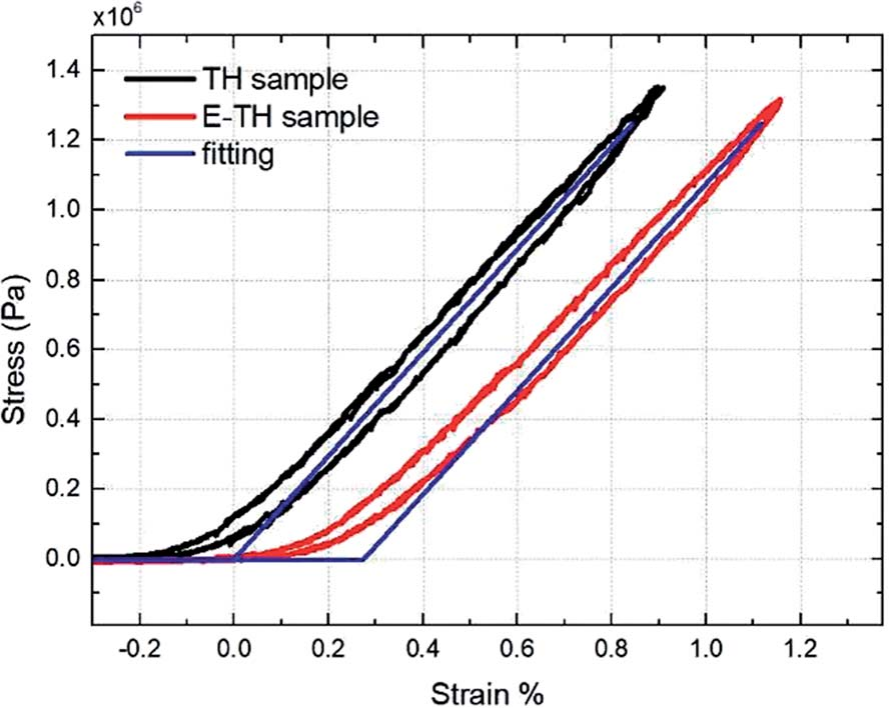
Stress *versus* strain: experimental curves for P(VDF-TrFE-CTFE) films prepared *via* both standard annealing (black) and electro-annealing (red), and fitting curves (blue).

To better understand the mechanical property of the terpolymer P(VDF-TrFE-CTFE), it is necessary to empirically determine its Young's modulus. The measurement was performed by recording the longitudinal tensile force under a given uniaxial displacement at 100 mHz. The 50 × 10 mm rectangular specimens with 90 μm-thick terpolymer films were used and a full description of the developed test bench was detailed in our previous works.^43^Fig. 3 shows that determining the slope of the stress-*versus*-strain curve, an estimation of the Young's modulus values can be obtained equal to 148 MPa for both TH sample and E-TH sample. Consequently, the electro-annealing method does not modify the mechanical property of polymer. This is consistent with values previously reported in the literature.^44,45^ A mechanical model was implemented by fitting it to the experimental curves as shown in Fig. 3 demonstrating good coherence.

## 4. Results and discussion

### 4.1 Morphological characterization

Three structural characterization steps were performed across different scales to infer improvements in electrical behavior of P(VDF-TrFE-CTFE) terpolymer to the ionic impurities polarization induced by the electro-thermal annealing (Subsection 3.1).

**Fig. 4:**
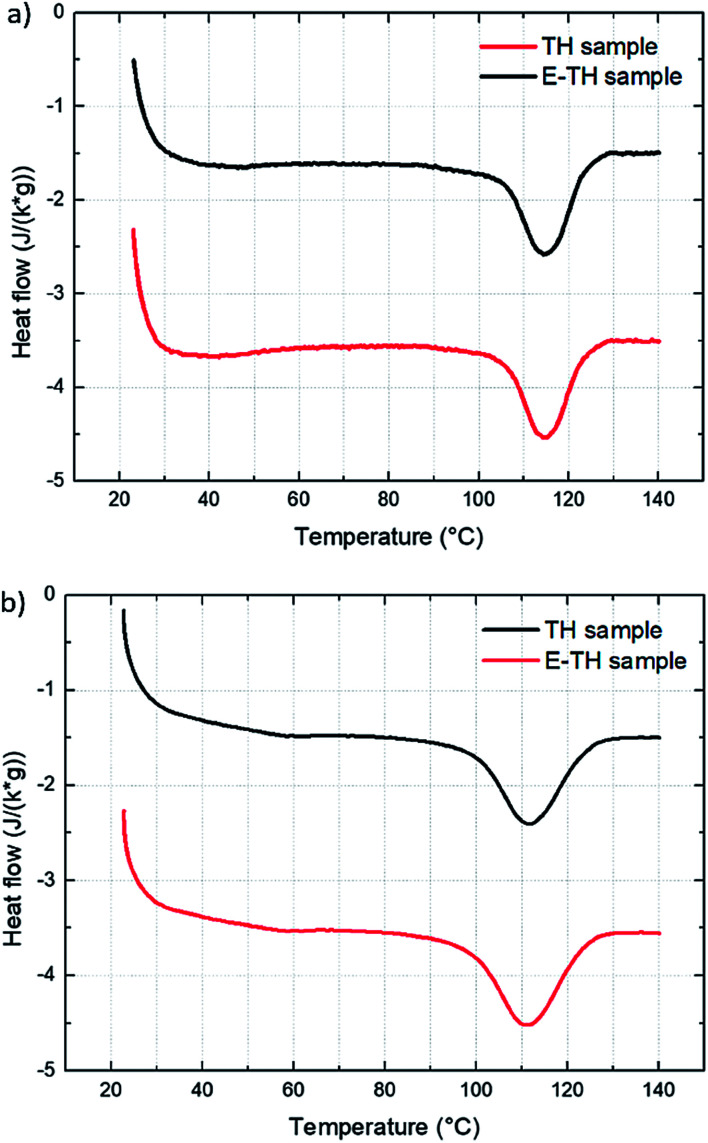
DSC thermographs for the two samples with two different heating rates: (a) 10 °C min^−1^ and (b) 20 °C min^−1^.

The DSC results (Fig. 4) confirmed no modifications in crystalline morphology from the two different annealing methods. The thermographs had no variation in melting peak position, width, or appearance of bimodal shapes. This result demonstrated that the electro-annealing did not introduce any change in crystal formation to the crystal size or crystal size distribution. Fig. 4a shows the thermograph for the two differently annealed samples with an heating rate of 10 °C min^−1^. Integration of melting peaks results in values of melting enthalpy of 12.2 J g^−1^ corresponding to 29% degree of crystallinity.^40^ To verify that the heating rate does not distort the measurements—especially in case of fast crystallization dynamic of material—thermographs with faster heating ramp (20 °C min^−1^) were taken as illustrated in Fig. 4b. There were no differences between samples. The results of thermal analysis are summarized in Table 1.

**Table 1:** DSC thermal analysis results of TH and E-TH samples measured with two different heating rates

	TH sample		E-TH sample	
	10 °C min^−1^	20 °C min^−1^	10 °C min^−1^	20 °C min^−1^
*T*_melting_ (°C)	114.6	114.3	114.3	114.0
Δ*H*_melting_ (J g^−1^)	12.2	13.4	12.1	14.1
FWHM (°C)	12.7	13.0	11.5	14.5
*χ*_c_%	29.3	31.9	28.8	33.6

**Fig. 5:**
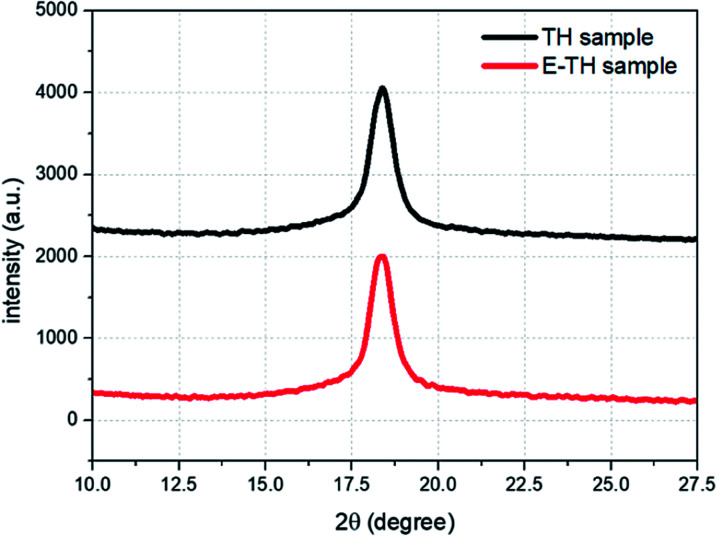
X-ray spectra for two annealed samples.

A deeper investigation of crystal structure was carried out based the XRD analysis. The diffraction spectra (Fig. 5) shows diffraction peaks perfectly superimposable for the two samples because there was no variation in peak position (2*θ* = 18.4°) or peak width (FWHM = 0.83°). Therefore, no modification in crystal lattice space was found confirming that the electro-annealing treatment did not alter the crystalline phase conformation. Similar to the DSC thermographs, the XRD diffraction peaks do not exhibit any variation in crystal size distribution for both samples.

**Fig. 6:**
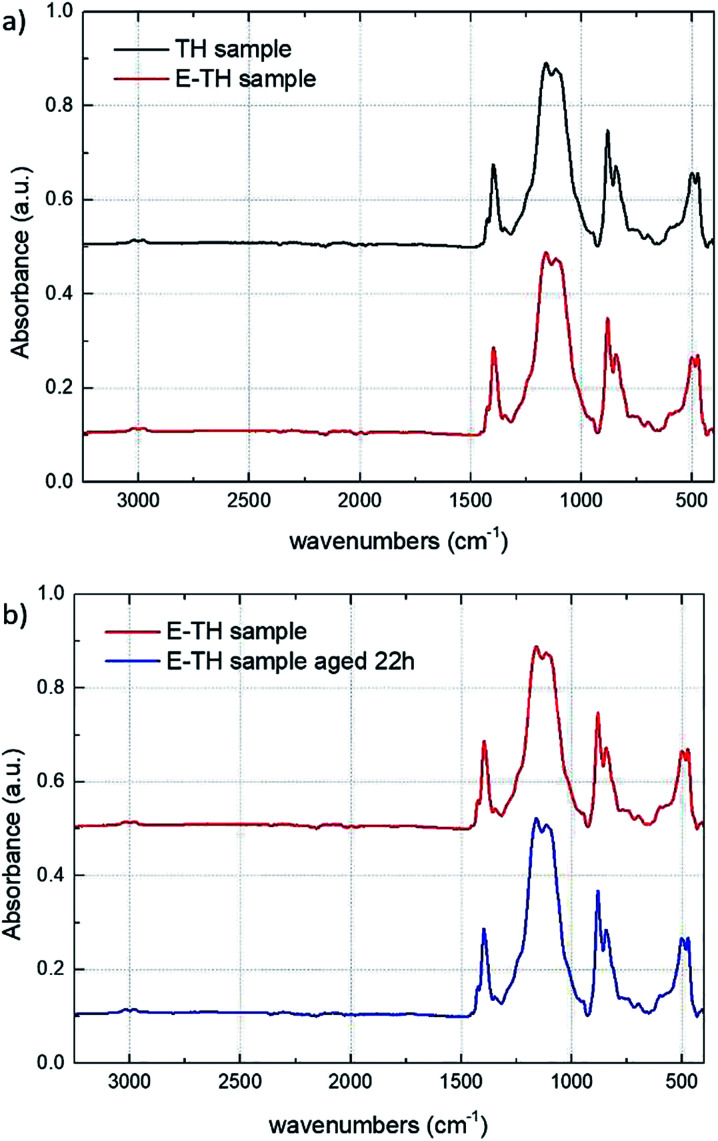
(a) IR spectra for TH and E-TH samples. (b) IR spectra for E-TH sample before and after 22 h of aging.

Fig. 6a depicts the ATR-IR spectra of the TH and E-TH samples based on two different annealing procedures. Both spectra were very similar affirming that the novel electro-annealing technique did not induce any structural modification to the P(VDF-TrFE-CTFE) films. These results are of particular interest and suggest that the improvements in electrical behavior of EAPs in terms of leakage current reduction and dielectric losses are mainly due to the interfacial polarization of ionic impurities. Fig. 6b shows IR analysis of the E-TH samples before and after 22 h of aging. Again, no structural modifications were observed.

### 4.2 High-voltage leakage current

**Fig. 7:**
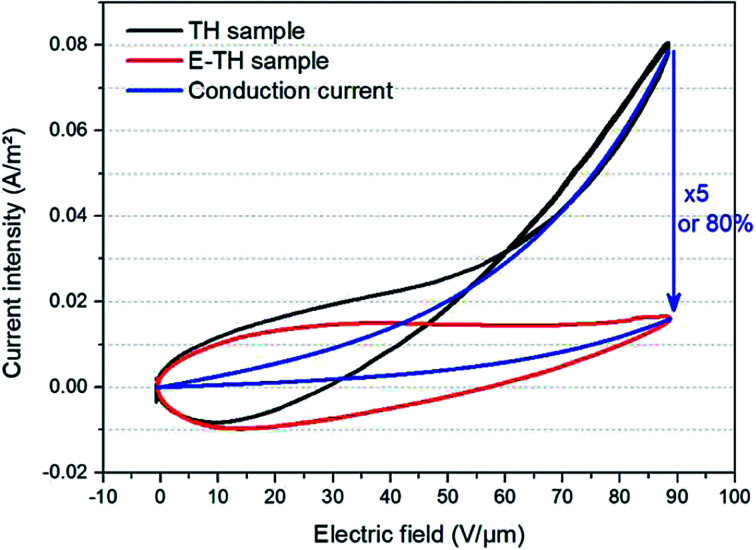
Current *versus* electric field for unipolar sinusoidal voltages. Black and red lines refer to experimental measurement of the TH-sample and E-TH sample, respectively. Blue curves represent the conduction current component modelled by eqn (3).

Fig. 7 depicts the experimental current under a unipolar sinusoidal electric field of 90 V μm^−1^ for the two different annealed samples. As expected, the conduction current density of the TH sample was strongly nonlinear as a function of the applied electric field—particularly under high voltage excitation where the curve tends to have an exponential trend. On the other hand, the E-TH sample has a much better linear behavior for the entire range of applied electric fields. This leads to a significantly reduced leakage current density. At 90 V μm^−1^, there is a huge decrease of 80% in the leakage current density corresponding to a five-fold lower value *versus* the TH sample.

The blue slope of Fig. 7 described the conduction (or leakage) current that can be estimated based on the following Hopping model:^46,47^2
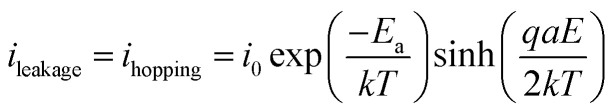
where *E*_a_ is the conduction mechanism activation energy related to the average trap depth, *k* and *T* are the Boltzmann constant and the temperature, respectively, *q* is the charge carrier charge, *a* is the average trap distance, and *E* is the external applied electric field.

For the sake of simplicity, the Hopping model can be rewritten as follows:3
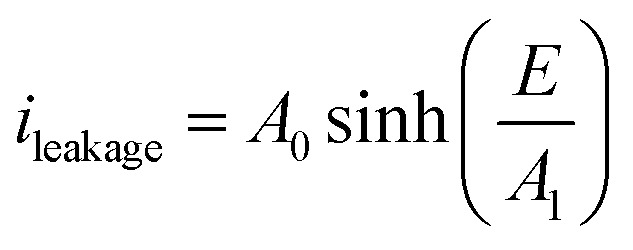
where the constant *A*_0_ and *A*_1_ are given by:4
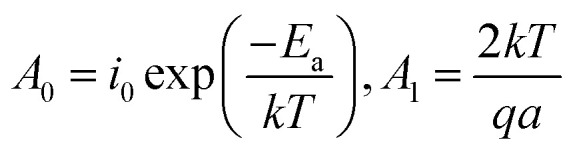


The evaluation of *A*_0_ and *A*_1_ parameters of each sample has been performed by fitting the curve of experimental current *versus* electric field. Table 2 summarizes the modeling results. As expected, only *A*_0_ parameters varied whereas *A*_1_ was constant for the both TH and E-TH terpolymers. This is consistent to previous works.^33,48–51^ Interestingly, *A*_0_ showed a 5-fold reduction for the E-TH sample with respect to the TH one, and this parameter is inversely proportional to the activation energy of conduction mechanism.

**Table 2:** Results of modeling hopping conduction current for different samples

	*A*_0_ (A)	*A*_1_ (A m^2^ V^−1^)	*a* (nm)	*A* ^TH^_0_/*A*^E-TH^_0_
TH sample	7.4 × 10^−3^	2.89 × 10^7^	0.89	
E-TH sample	1.49 × 10^−3^	2.89 × 10^7^	0.89	5

Accordingly, the fitting results revealed that the proposed electro-thermal annealing did not cause any morphological modification of polymer amorphous phase;^52^ the remarkable 80% decrease in leakage current was inferred to a 5-fold increased energy barrier of the electronic conduction mechanisms.

### 4.3 Dielectric losses

**Fig. 8:**
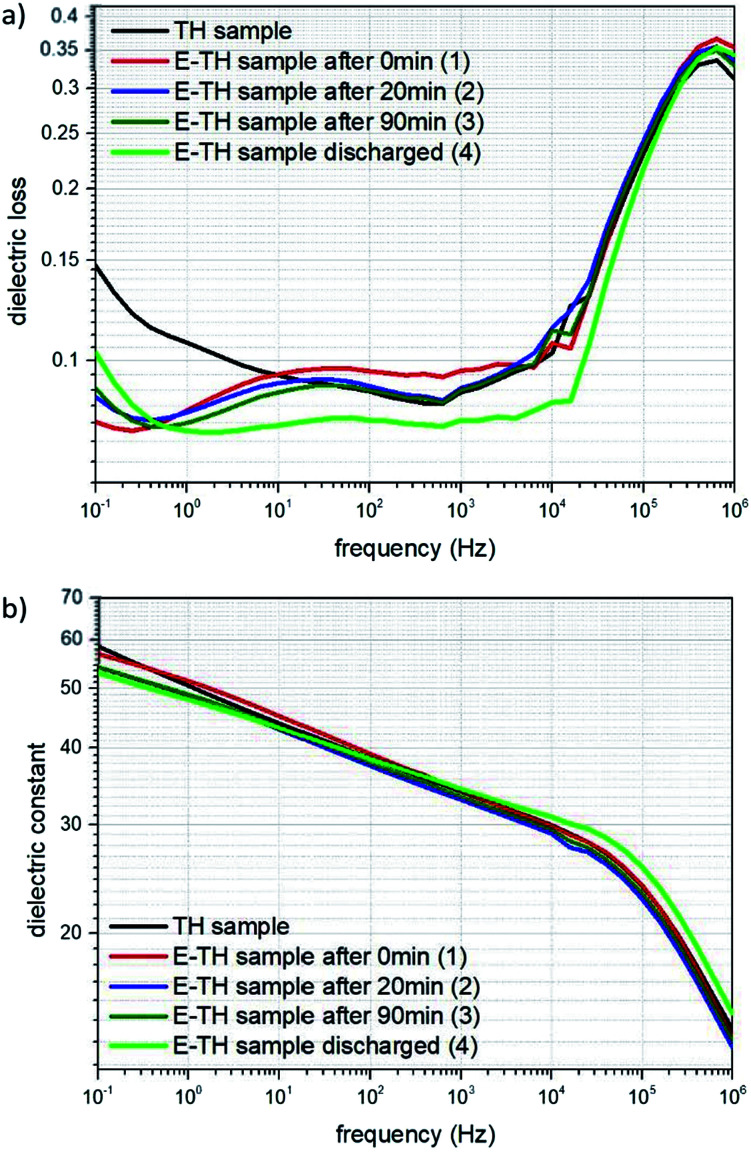
(a) Dielectric losses and (b) dielectric constant for TH (black line) and E-TH sample just after the electro-thermal treatment (red line) and after different times from the treatment (blue and dark green lines). The discharged sample (light green line) refers to the reference sample in which residual injected charges are totally evacuated.

The broadband dielectric spectroscopy was studied at low frequency ranges comprising dielectric loss (the so-called tan(*δ*)) and dielectric constant (*i.e.* relative permittivity). This data helps compare the level of ionic conductivity for different annealed samples (Fig. 8). For this study, the TH sample as well as the E-TH film with four different process were investigated including 1, 2 and 3 after the electro-thermal annealing process of 0 min, 20 min, 90 min, respectively. This details the instability effect due to superficial charge residues. Sample 4 was E-TH discharged sample obtained by being simultaneously short-circuited (*i.e.* its two electrodes connected together) and heated in the oven for an additional 90 min at 100 °C to fully evacuate the remaining injected charges.

The spectrum in Fig. 8a shows that under low frequency of 100 mHz, the dielectric losses tan(*δ*) of all E-TH samples dropped dramatically achieving a value of 0.078 instead of 0.15 as in the case of the conventional TH sample. This property leads to substantially decreased dielectric losses of almost 50%. This was caused by limited ionic conductivity of the E-TH samples. Similar results under very low frequencies were obtained for the E-TH discharged sample. Nonetheless, at higher frequency ranges (*i.e.*, around 10^6^ Hz where the relaxation peak occurs relating to the polymer chain α-relaxations (so-called dielectric glass transition)) there were no difference in dielectric losses behavior of the all samples. No structural modifications were observed regardless of which thermal treatment was selected: standard or electro-annealing process. These characteristics lead to an unchanged dielectric constant in the terpolymer films across the entire frequency range as demonstrated in Fig. 8b.

**Fig. 9:**
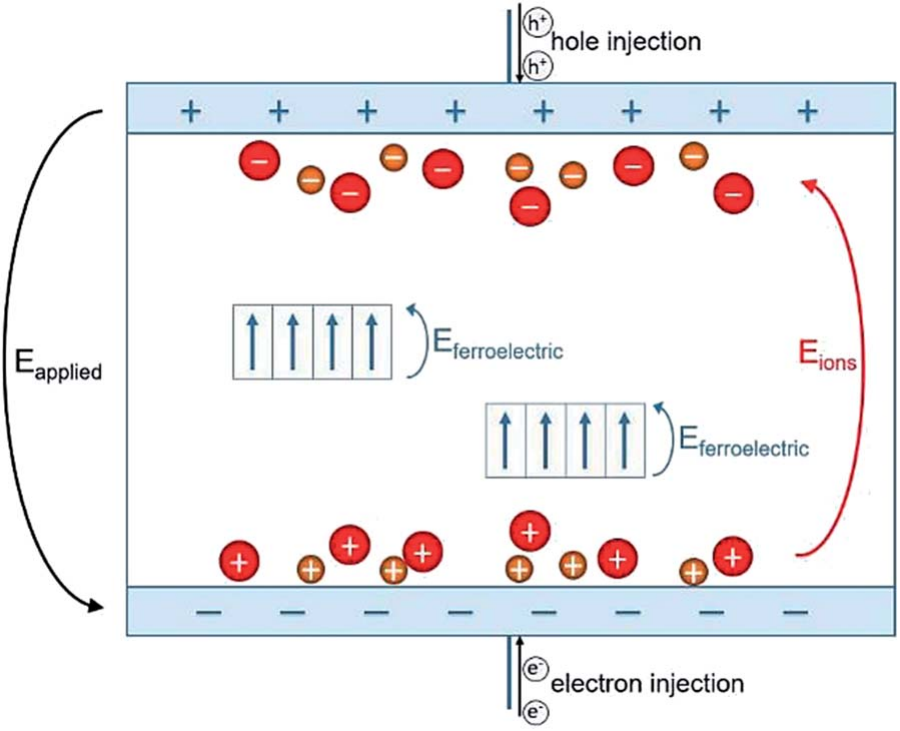
Representation of polarization mechanisms in terpolymers for a positive applied electric field. The red and orange circles represent the ionic impurities; blue rectangle and inner arrows refer to ferroelectric domain dipoles.

To conclude, high-voltage electronic conduction can be controlled *via* a simple polarization of ionic species presented in the polymer matrix. The polymerization agents used in the polymer synthesis of P(VDF-TrFE-CTFE) terpolymer contain ionic impurities that, although only on the order of ppm, contribute to interfacial polarization.^24,53–55^ These ionic impurities represent heterocharges that can be driven toward the oppositely charged electrode and accumulate under a constant DC voltage excitation.^56^ A schematic representation of this ionic polarization process is shown in Fig. 9.

In our case, during the electro-thermal annealing, the polarization process of ionic species was further promoted by the high temperatures at which the ion mobility in the polymer matrix is enhanced^24^ especially at longer times. Subsequently, the ionic charges turned out to be “locked” at the polymer/electrode interface during the cool-down step to room temperature when the molecular mobility was strongly reduced. This formed an electric double layer that had a dual effect on dielectric loss. First, their contribution to ionic conductivity was strongly reduced because the ionic species were constrained at the interface.^53^ This was observable in dielectric losses spectrum at low-frequency (Fig. 8a). Secondly, the accumulation of hetero-charges build up a local electric field (renamed *E*_ions_) opposed to the applied electric field that lead to a smaller internal electric fields and thus reduced driving force for electronic conduction^57,58^ as measured at high-voltage. This was confirmed by the modeled increased activation energy for electronic conduction in Subsection 4.2.

### 4.4 Polarization measurements

**Fig. 10:**
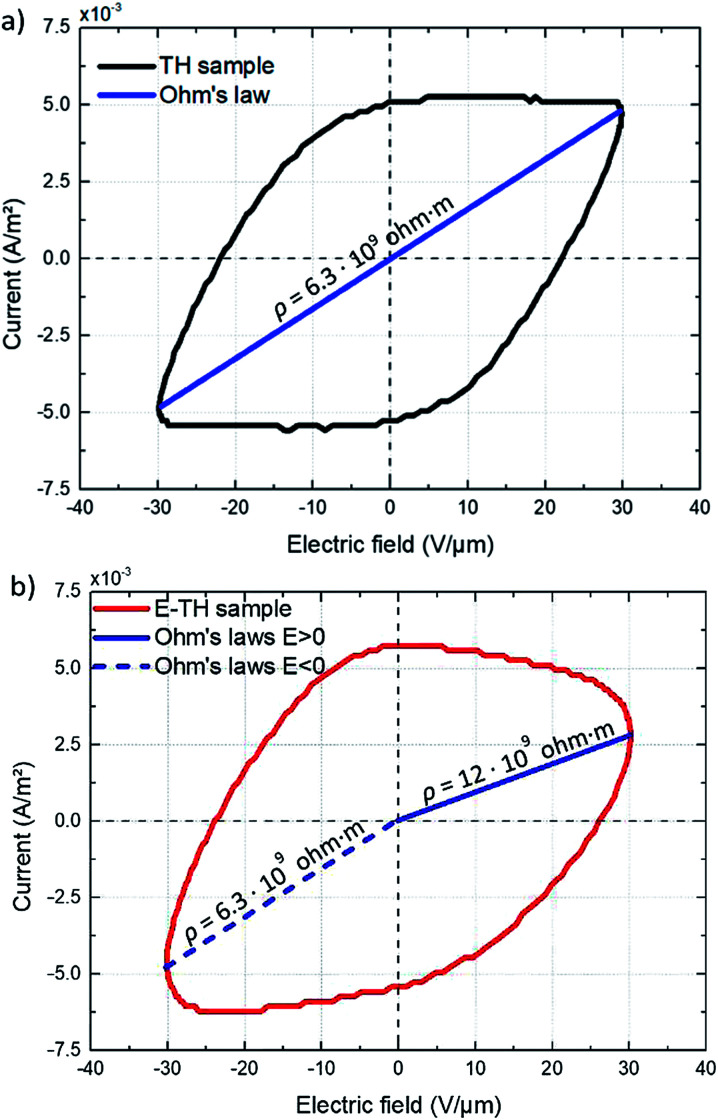
Current *vs.* AC electric field for (a) TH sample and (b) E-TH sample. Blue lines represent the conduction of Ohm's law, and slope values are dedicated to the polymer volume resistance.

Fig. 10 displays the experimental current *versus* electric field for the two different annealed samples using a bipolar AC sinusoidal voltage at 50 mHz. Fig. 10a shows that the resulting current of the TH sample has a typical feature of a relaxor-ferroelectric material^59^ exhibiting a perfectly symmetrical and linear relationship under voltages of *E*_peak–peak_ = ±30 V μm^−1^. This resistive behavior can be described *via* Ohms's law:5
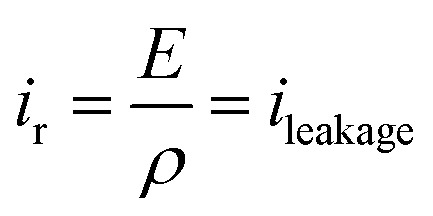
Here, *ρ* is the volume resistivity of polymer corresponding to the inverse of the slope (blue line) in Fig. 10a.

The *ρ* of the TH samples was estimated to be 6.3 × 10^9^ ohm m. The E-TH sample (Fig. 10b) exhibited an asymmetric current that depends on the sign of the applied electric field. Based on the fitting results of eqn (5), two different values of the polymer volume resistivity were obtained, *i.e. ρ* = 6.5 × 10^9^ ohm m for *E* < 0, and *ρ* = 12.0 × 10^9^ ohm m for *E* > 0. Interestingly, the resistivity value of the E-TH sample was similar to that of the TH sample under a negative input voltage—this was not the case for the positive electric field.

According to the observation drawn in the previous Subsection 4.3, such an asymmetrically resistive behavior to electronic conduction of the E-TH sample is due to the electric double layer formed at the polymer/metal interfaces by ions polarization achieved during the electro-thermal annealing. Under appliance of positive electric field, as represented in Fig. 9, the ionic charges accumulated at the polymer/electrode interfaces during the electro-thermal annealing are oppositely charged with respect to the adjacent electrode. The so-built electric double layers can represent a limitation for injection of homocharges^56^ (*i.e.* electrons and holes) resulting in the reduced leakage current through the sample,^60,61^ which was not the case for the standard annealed TH sample. Moreover, overall internal electric field turns out to be reduced by the interfacial polarization of the ionic hetero-charges. Anions and cations separation builds up a local electric field that, as depicted in Fig. 9, opposes to the positive applied electric field leading to lower driving forces for electron motion,^57,58^ reducing the electronic conduction through the sample.

Consequently, using positive voltage excitation nearly doubled the resistivity of the E-TH sample resulting in considerably improved electrical properties like reduced leakage current as well as dielectric losses. In case of negative voltage appliance, the electrode polarity was reversed; thus, the electric double layer no longer holds resulting in unchanged resistivity values that is similar to the one of TH sample.

Fig. 10 highlights that the experimental current corresponding to a total intensity (*i*_tot_) consists of a capacitive current (*i*_cap_) and a conduction current (*i*_leakage_); the latter is given by eqn (5). Subsequently, the capacitive current can be easily estimated (eqn (6)) by subtracting the leakage current from the total measured current.

6 *i*_cap_ = *i*_tot_ − *i*_leakage_

Eventually, the electric displacement *D*(*E*) is determined by time integration of the capacitive current density that is written by:7
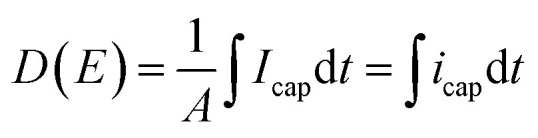


**Fig. 11:**
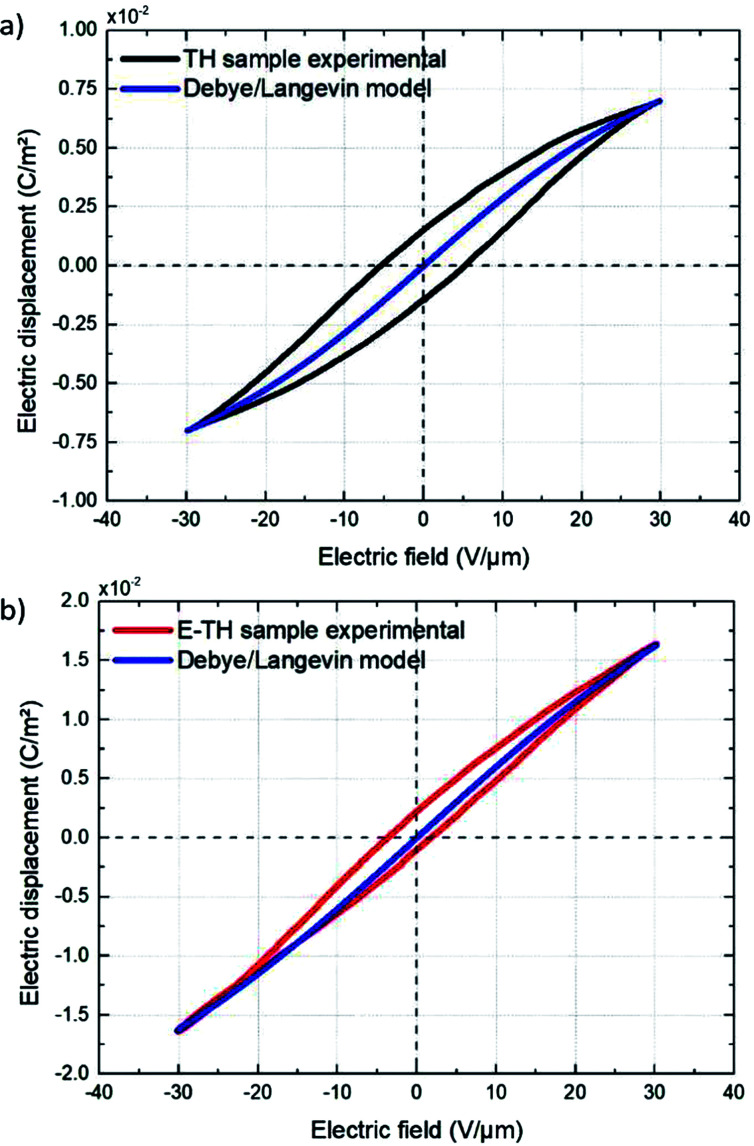
Experimental electric displacement *vs.* electric field curves for (a) TH sample and (b) E-TH sample. Blue lines represent the polarization calculated from the Debye/Langevin model.

Fig. 11 displays the electric displacement *versus* the applied electric field for the TH and E-TH samples. As expected, the polarization loop *D*(*E*) of the E-TH sample was asymmetric—this is the opposite of the case of the TH sample. This result is consistent with the previous analysis relating to the leakage current behavior.^33^

The electric displacement loop of the two samples were then fitted by the theoretical Debye/Langevin model. Further details of this approach have been fully described in our previous works.^33,62,63^ The two-component Debye/Langevin formalism considers the capacitive current *i*_cap_ to constitute the sum of contributions generated by the dielectric response of two inherent morphological phases (*i.e.* crystal and amorphous) present in the semi-crystalline P(VDF-TrFE-CTFE):

8 *i*_cap_ = *i*^crystal^_cap_ + *i*^amorph^_cap_

where *i*_cap_^*θ*^ (*θ* denotes crystal or amorphous) is calculated as:9
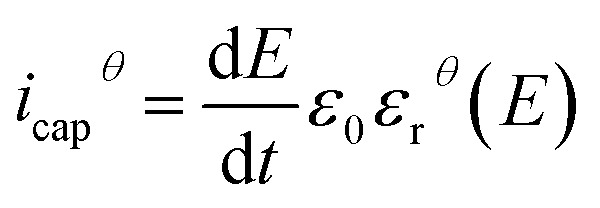


The relative dielectric permittivity *ε*_r_^*θ*^(*E*) can be estimated based on the following formula:10
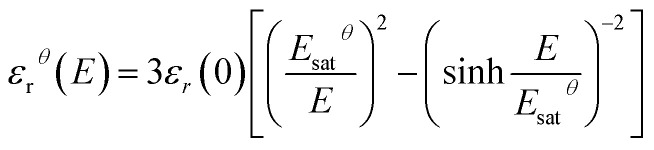
where *E*_sat_ is the saturation electric field of the relative material phase.

The experimental polarization curves as well as the corresponding fitting models are depicted in Fig. 11. The fitting results are in Table 3:

**Table 3:** Fitting results based the Debye/Langevin model

	TH sample		E-TH sample	
	*ε* ^*E*=0^_r_	*E*_sat_ (V μm^−1^)	*ε* ^*E*=0^_r_	*E*_sat_ (V μm^−1^)
Crystal	59	13	59	20
Amorphous	10	400	10	400

**Fig. 12:**
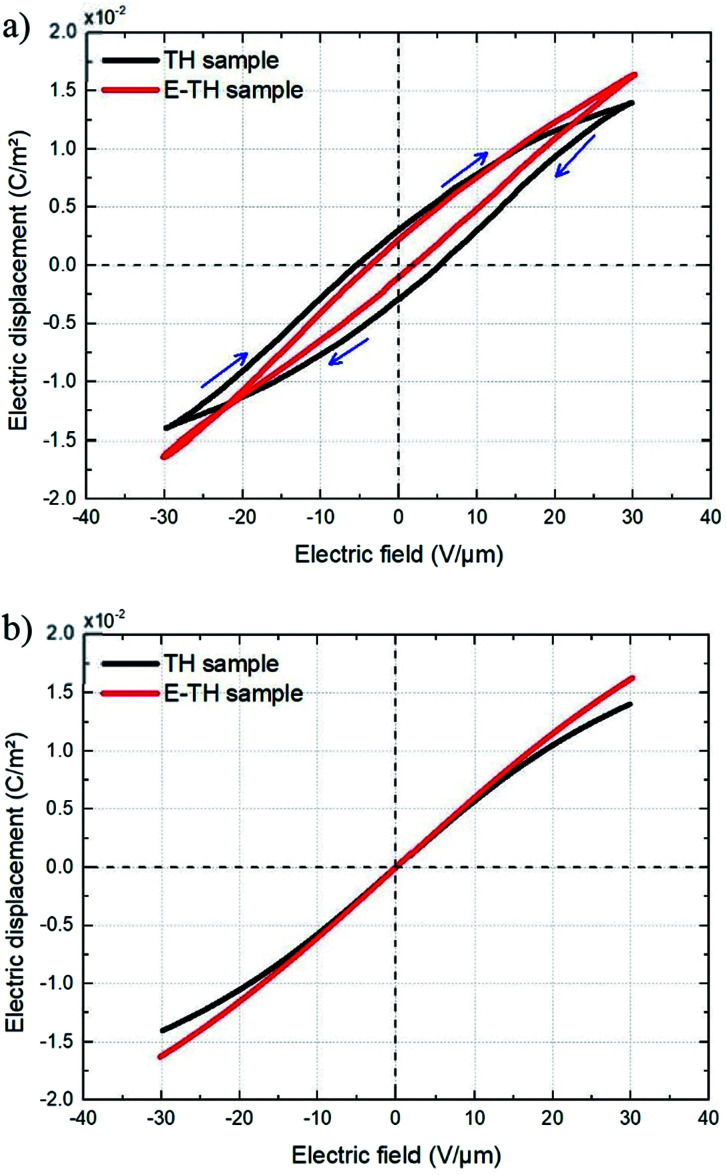
(a) Superposition of experimental electric displacement curves and (b) modeled polarization for the two samples. Direction of applied electric field is indicated by blue arrows.

The Debye/Langevin fitting result of the TH and E-TH samples shows that their intrinsic dielectric permittivity is identical in both the crystal and amorphous phases in contrast to the saturation electric field that is higher for the E-TH sample. For a better comparison, experimental loops and modeling curves for the two samples are superimposed in Fig. 12; characteristic polarization parameters are listed in Table 4.

**Table 4:** Electric displacement curve parameters

	TH sample		E-TH sample	
	*E* < 0	*E* > 0	*E* < 0	*E* > 0
*P*_max_ (C m^−2^)	1.40 × 10^−2^	−1.40 × 10^−2^	1.65 × 10^−2^	−1.64 × 10^−2^
*P*_remnant_ (C m^−2^)	3.02 × 10^−3^	−3.03 × 10^−3^	2.10 × 10^−3^	−1.00 × 10^−3^

The relevant asymmetry in the electric displacement loop of the E-TH sample is clearly observed; the absolute value of its remnant polarization under a positive electric field is two-fold lower than under a negative input excitation. This effect is largely caused by the non-relaxed ionic polarization (*E*_ions_ in Fig. 9) that counterbalances the intrinsic terpolymer remnant polarization induced by ferroelectric domains.

### 4.5 Electrical aging

**Fig. 13:**
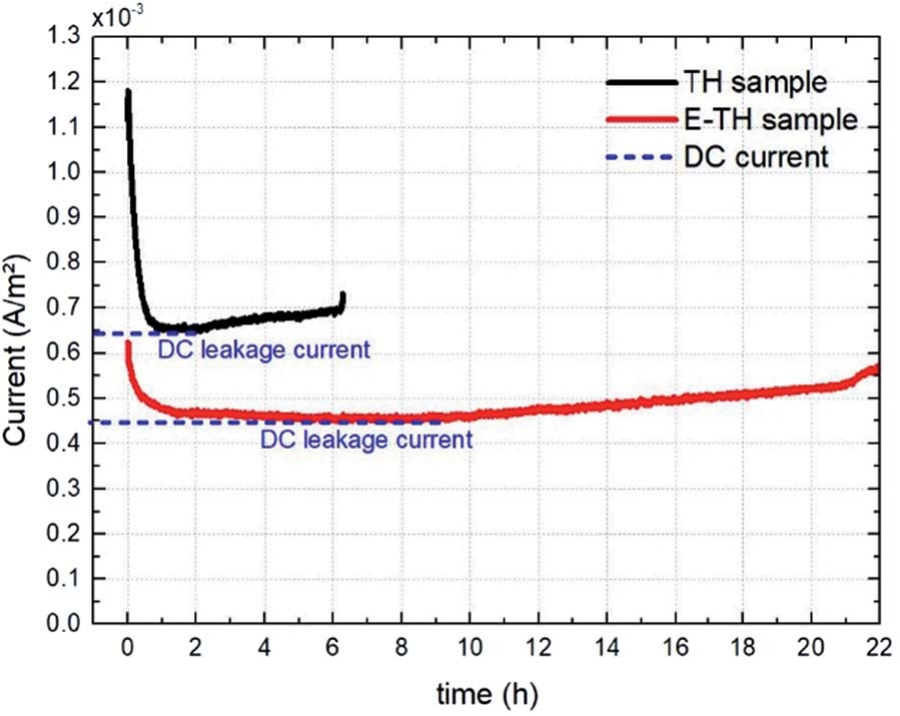
Current density *vs.* aging time for one pair of specimens including the TH sample and the E-TH sample. Dashed blue lines represent the level of leakage current density.

For a more reliable assessment of the effect engendered by the electro-thermal annealing treatments, the resulting current density of both TH and E-TH polymers was observed during a long-term experiment (22 hours) under a unipolar sinusoidal electric field of 40 V μm^−1^ at 100 mHz. The aging test evolution in Fig. 13 revealed that the DC current density level of the E-TH sample was clearly lower than the TH sample confirming the markedly enhanced electrical properties (reduced leakage current and dielectric losses) for the new process material.

Fig. 13 shows that the current *versus* time measurement. This measurement was characterized by an initial transitory regime that represents the capacitive and ionic drift current components due to dipole polarization and ionic polarization, respectively. Next, a steady regime occurred that was merely related to the DC conduction effect. At longer testing times, the current intensity again became unstable and began to increase. This final regime related to a run-away process due to the self-heating of polymer created by electric conduction in a thermally-activated mechanism.^29^ This mechanism corresponds to the material degradation phase.

It has been observed that the E-TH film leads to a drastically decreased DC leakage current: the value dropped to 4.5 mA m^−2^ as opposed to 6.5 mA m^−2^ for the standard material. This result demonstrates a significant improvement in resistive properties of around 30% for the novel developed polymer. Moreover, the last regime of the aging experiment showed that the E-TH sample had current-induced self-heating effects after 10 hours of electrical solicitation. This effect occurred after only 2 h of testing for the standard TH sample. Similar behavior was observed where the E-TH polymer led to 4-fold longer time-to-breakdown as opposed to the standard sample. In conclusion, the E-TH polymer is clearly much more resistant than the conventional one—particularly under a high voltage excitation.

### 4.6 Actuation performance

**Fig. 14:**
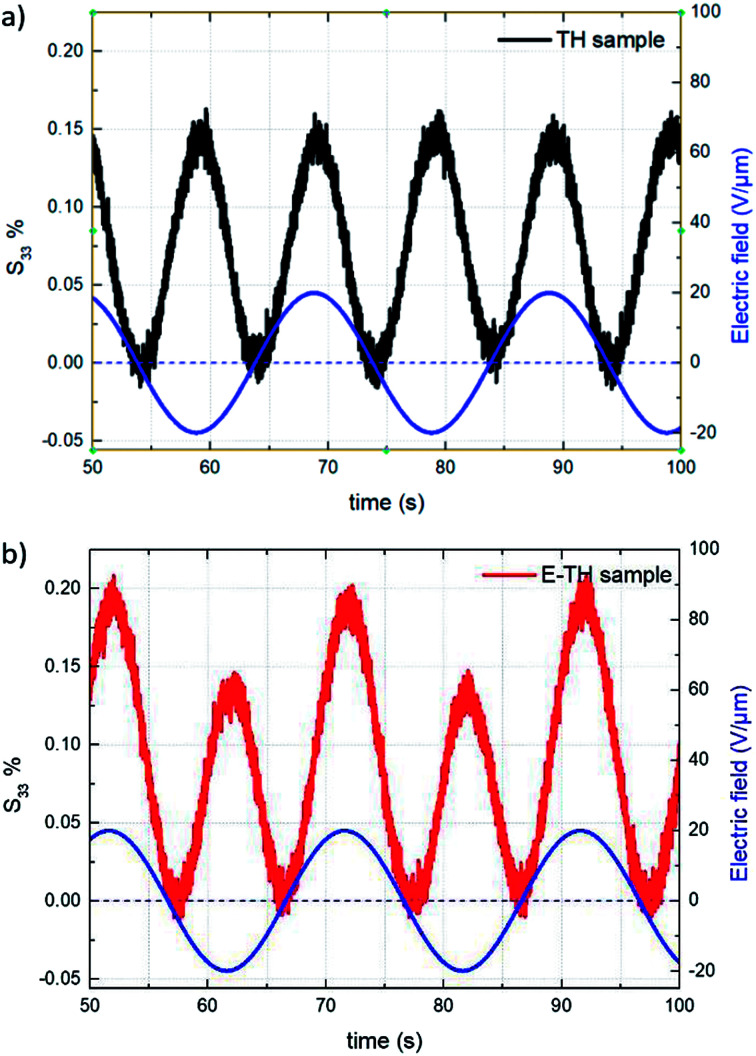
Strain *S*_33_*vs.* time under 20V μm^−1^ sinusoidal AC electrical input for (a) TH sample and (b) E-TH sample. Blue lines correspond to the applied electric field.

This subsection compares the electromechanical performance of the E-TH and TH samples under bipolar AC voltage of 50 mHz frequency and 20 V μm^−1^ amplitude. The empirical longitudinal strain *S*_33_*versus* electric field of both EAPs are illustrated in Fig. 14. Once again, the experimental results highlight two outstanding features related to the new annealed material, *i.e.* its enhanced actuation performance and its asymmetric mechanical response to the bipolar electrical input. Indeed, under negative voltage excitation, the longitudinal strain *S*_33_ of the E-TH sample is identical to the standard sample, and their values are 0.15%. This is in contrast to that with positive applied voltage where the E-TH polymer gives rise to a significant increase of 30% in electromechanical activity, *i.e.*, the resulting strain *S*_33_ increased from 0.15% to 0.2%. Such an improvement nicely fits the behavior of the DC leakage current whose value also decreased by approximately 30% as described in Section 4.5. Consequently, the electromechanical performances of EAPs truly reflects the polarization response confirming the impact of electro-annealing thermal process to the intrinsic electrical properties of polymers in terms of leakage current and dielectric losses.

To better assess the actuation performance of the EAPs, we provide here mathematical expressions of the electrostrictive coefficient (*M*_33_) and the electromechanical coupling constant (*Q*_33_).

The *Q*_33_ is defined by the relationship between the longitudinal strain *S*_33_ and the sample macroscopic polarization *D*(*E*) based on the following formula:^42,62,64^

11 *S*_33_ = *Q*_33_*D*(*E*)^2^

Hence, *Q*_33_ can be expressed as a function of the Young's modulus *Y* and the dielectric permittivity *ε*:12
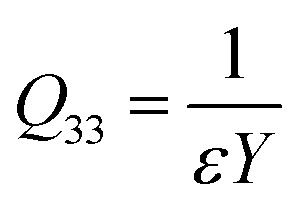
where *ε* can be derived either by the experimental measurements of electric displacement (Fig. 11) or by the Langevin/Debye extrapolation of eqn (8)–(10) resulting in two possible models of the electric-field induced longitudinal strain:^62^13
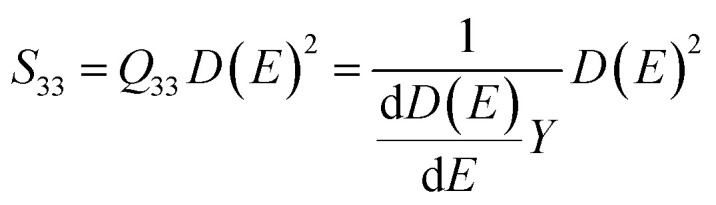
14
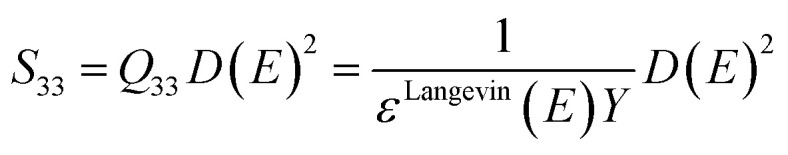


Regarding the electrostrictive coefficient (*M*_33_), the quadratic relationship between the longitudinal strain *S*_33_ and the applied electric field *E* allows to identify its value:15
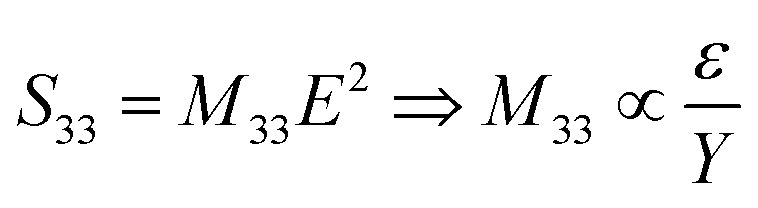


Importantly, the Young's modulus used in eqn (12)–(15) formally refers to the polymer's mechanical behavior under compression strains. In this study, it can be assumed that the Young's modulus is unchanged in both compression and tensile configurations considering the small deformation in the linear elasticity area and the low crystallinity of terpolymer.

**Fig. 15:**
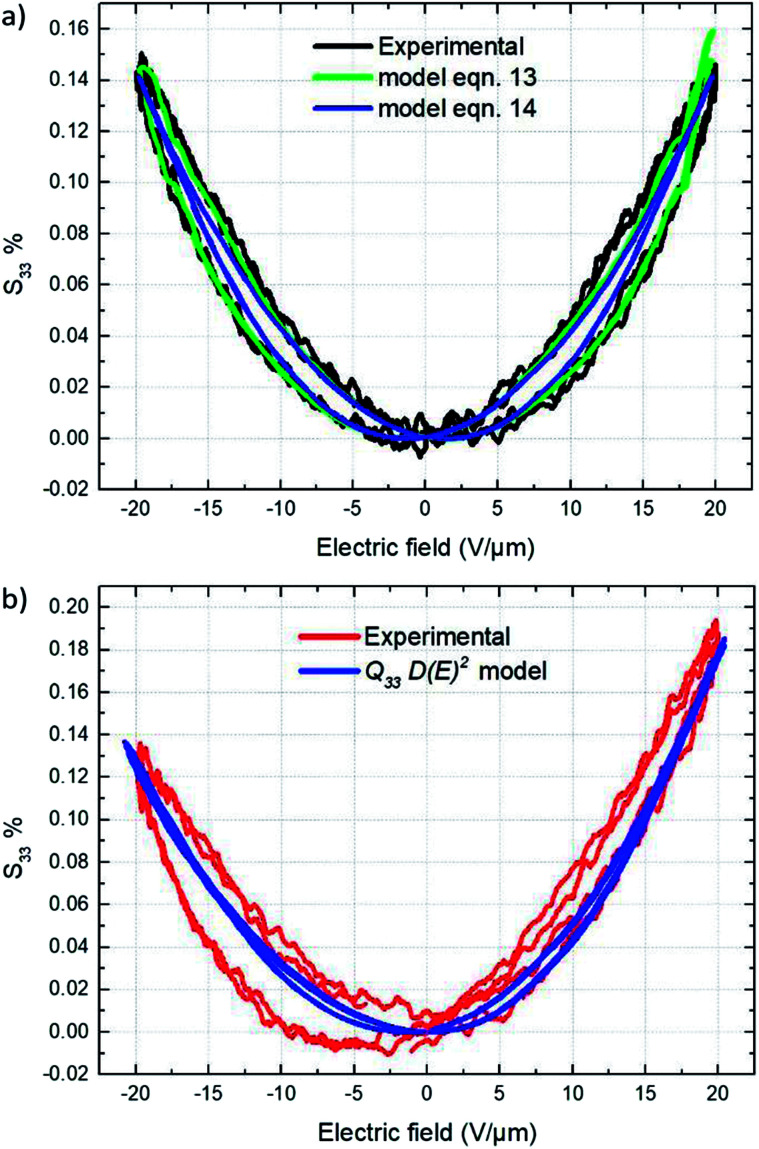
Electromechanical measurements and fitting results of (a) TH sample and (b) E-TH sample.

Fig. 15a depicts the experimental electromechanical characterization as well as the corresponding theoretical model of the conventional TH materials. Longitudinal strain *S*_33_*versus* applied electric field curve shown in Fig. 15a represents the typical electro-mechanical behavior of a ferro-relaxor material^65,66^ where the residual or remnant polarization, due to the not completely relaxed ferroelectricity, induces hysteresis in the longitudinal strain *S*_33_*versus* applied electric field curves.^67,68^ Both theoretical models (13) and (14) perfectly fit the experimental curves. Eqn (12) with a Young's modulus of 148 MPa (Subsection 3.4) yielded an estimate of 13.2–14.5 m^4^ C^−2^ for the electromechanical coupling constant *Q*_33_ under an applied electric field varying from 0 to 20 V μm^−1^.

The electrostrictive coefficient *M*_33_, as defined by eqn (15), turns out to be not constant and dependent on the applied electric field. More precisely, the value of permittivity tends to somewhat decrease with the input electric field due to dipole saturation. However, this variation in permittivity is relatively low and it can be negligible under electric field not excess 20 V μm^−1^. Indeed, as observed in Fig. 12b, the dielectric response of both TH and E-TH samples is quasi-linear at such an input voltage range. This leads to unchanged electrostrictive coefficient *M*_33_ for an electric field range ≤ 20V μm^−1^.

Accordingly, from eqn (15), the estimate values of the quasi-constant electrostrictive coefficient *M*_33_ were 3.6 × 10^−18^ m^2^ V^−2^ and 4.4 × 10^−18^ m^2^ V^−2^ for the TH and E-TH polymers, respectively. The estimates of both *Q*_33_ and *M*_33_ were consistent with literature reports for polar electrostrictive polymers.^13,17,18,62^

As demonstrated in Subsection 4.4, the Debye/Langevin model was not valid for the E-TH sample because of its asymmetrical electrical polarization that was inverse to the standard TH material. Indeed, such asymmetrical dipolar properties cannot be correlated by a unique model parameter that describes the strain response under both negative and positive voltages. Thereby, the experimental strain of the E-TH polymer were fitted by eqn (11) with different parameters depending on the sign of the input excitation. Fig. 15b shows the empirical curves of the E-TH sample under negative electric fields. These curves were well-fitted based on the same values of *M*_33_ and *Q*_33_ used for the TH sample. Nonetheless, under positive electrical inputs, the E-TH polymer leads to increased electrostrictive coefficient *M*_33_ of 22% and enhanced electromechanical coupling constant *Q*_33_ of 14% with respect to the former TH terpolymer. For a better comparison, Table 5 summarizes the values of both coefficients characterizing the electromechanical performances of the E-TH and TH materials.

**Table 5:** Electromechanical performance

	TH sample	E-TH sample
*M*_33_ (m^2^ V^−2^)	3.6 × 10^−18^	4.4 × 10^−18^
*Q*_33_ (m^4^ C^−2^)	13.2–14.5	16.5

## 5. Conclusions

This work proposed an innovative processing method to successfully decrease the leakage current as well as dielectric losses of EAPs under high voltage. The additional fabrication step consists of annealing the semicrystalline P(VDF-TrFE-CTFE) terpolymer under a constant DC voltage, *i.e.* the so-called electro-thermal annealing. The experimental results demonstrated that such novel processing leads to a drastically reduced leakage-current of 80% under low frequency excitation making it possible to enhance the lifetime of EAPs. Structural and electrical characterization revealed that the new electro-annealing procedure did not alter the polymer morphology; rather, it favored the interfacial polarization process by reducing the contribution to ionic conductivity while limiting the presence of ionic impurities in the polymer matrix. Furthermore, the electromechanical characterization confirmed an improvement in actuation performance of 30% that matched the decreased leakage current density reflecting a relationship between the dissipated energy due to the driven high-voltage conduction and electromechanical coupling of EAPs.

## Conflicts of interest

There are no conflicts to declare.

## Supplementary Material

## References

[cit1] Ganet F., Le M. Q., Capsal J. F., Lermusiaux P., Petit L., Millon A., Cottinet P. J. (2015). Sci. Rep..

[cit2] Kassa H. G., Nougaret L., Cai R., Marrani A., Nysten B., Hu Z., Jonas A. M. (2014). Macromolecules.

[cit3] Choi S. T., Kwon J. O., Bauer F. (2013). Sens. Actuators, A.

[cit4] Zhang S., Klein R. J., Ren K., Chu B., Zhang X., Runt J., Zhang Q. M. (2006). J. Mater. Sci..

[cit5] Meijer K., Rosenthal M. S., Full R. J. (2001). Proc. SPIE.

[cit6] Capsal J. F., Galineau J., Lallart M., Cottinet P. J., Guyomar D. (2014). Sens. Actuators, A.

[cit7] Della Schiava N., Thetpraphi K., Le M. Q., Lermusiaux P., Millon A., Capsal J. F., Cottinet P. J. (2018). Polymers.

[cit8] Le M.-Q. , Marrani A., Pedroli F., Tauban M., Cottinet P.-J., Sanseau O. and Capsal J.-F., Electroact. Polym. Actuators Devices XX, 2018, vol. 1059413, p. 36

[cit9] Zhu L. (2014). J. Phys. Chem. Lett..

[cit10] Ganet F., Le M.-Q., Capsal J. F., Gérard J. F., Pruvost S., Duchet J., Livi S., Lermusiaux P., Millon A., Cottinet P.-J. (2015). Sens. Actuators, B.

[cit11] Pelrine R., Kornbluh R., Pei Q., Joseph J. (2000). Sci. 80.

[cit12] Su J., Moses P., Zhang Q. M. (1998). Rev. Sci. Instrum..

[cit13] Eury S., Yimnirun R., Sundar V., Moses P. J., Jang S. J., Newnham R. E. (1999). Mater. Chem. Phys..

[cit14] Xia F., Cheng Z., Xu H., Li H., Zhang Q., Kavarnos G. J., Ting R. Y., Abdul-Sedat G., Belfield K. D. (2002). Adv. Mater..

[cit15] Chu B., Zhou X., Ren K., Neese B., Lin M., Wang Q., Bauer F., Zhang Q. M. (2006). Sci. 80.

[cit16] Yang L., Li X., Allahyarov E., Taylor P. L., Zhang Q. M., Zhu L. (2013). Macromolecules.

[cit17] Liu Q., Capsal J., Richard C. (2015). International Journal of Chemical, Molecular, Nuclear, Materials and Metallurgical Engineering.

[cit18] Yin X., Liu Q., Galineau J., Cottinet P. J., Guyomar D., Capsal J. F. (2016). Eur. Polym. J..

[cit19] Krebs F. C. (2009). Sol. Energy Mater. Sol. Cells.

[cit20] Naber R. C. G., Asadi K., Blom P. W. M., De Leeuw D. M., De Boer B. (2010). Adv. Mater..

[cit21] Naber R. C. G., Tanase C., Blom P. W. M., Gelinck G. H., Marsman A. W., Touwslager F. J., Setayesh S., de Leeuw D. M. (2005). Nat. Mater..

[cit22] Ling Q. D., Liaw D. J., Zhu C., Chan D. S. H., Kang E. T., Neoh K. G. (2008). Prog. Polym. Sci..

[cit23] Cai R., Kassa H. G., Haouari R., Marrani A., Geerts Y. H., Ruzié C., Van Breemen A. J. J. M., Gelinck G. H., Nysten B., Hu Z., Jonas A. M. (2016). Nanoscale.

[cit24] Huang H., Chen X., Yin K., Treufeld I., Schuele D. E., Ponting M., Langhe D., Baer E., Zhu L. (2018). ACS Appl. Energy Mater..

[cit25] Li H., Li Z., Xu Z., Lin F., Wang B., Li H., Zhang Q., Wang W., Huang X. (2014). IEEE Trans. Plasma Sci..

[cit26] Hua L., Fuchang L., Heqing Z., Ling D., Yongxia H., Zhonghua K. (2009). IEEE Trans. Magn..

[cit27] Handb. Low High Dielectr. Constant Mater. Their Appl., ed. H. S. Nalwa, Academic Press, Burlington, 1999, pp. xix–xx

[cit28] Sarjeant W. (1990). IEEE Trans. Electr. Insul..

[cit29] Fothergill J. C. , Electrical Degradation and Breakdown in Polymers, Institution of Engineering and Technology, 1992

[cit30] Grossiord N., Kroon J. M., Andriessen R., Blom P. W. M. (2012). Organic Electronics: Physics, Materials, Applications.

[cit31] Reed C. W., Cichanowski S. W. (1994). IEEE Trans. Dielectr. Electr. Insul..

[cit32] Brinati G. , Marrani A. and Goffaux B., Vinylidene fluoride and trifluoroethylene containing polymers, *US Pat.*, No. 8,575,286, 5 Nov. 2013

[cit33] Pedroli F., Marrani A., Le M.-Q., Froidefond C., Cottinet P.-J., Capsal J.-F. (2018). J. Polym. Sci., Part B: Polym. Phys..

[cit34] Liu Q., Yin X., Richard C., Capsal J. F. (2016). J. Polym. Sci., Part B: Polym. Phys..

[cit35] Kao K. C. , in Dielectr. Phenom. Solids, ed. K. C. Kao, Academic Press, San Diego, 2004, pp. 515–572

[cit36] Suyama T. S. S., Okamoto A. (1988). ECS J. Solid State Sci. Technol..

[cit37] Tu D. M. , Wang X. and Kao K. C., in Proc. IEEE Conf. Electr. Insul. Dielectr. Phenom. (CEIDP '93), 1993, pp. 550–555

[cit38] Liufu D., Wang X. S., Tu D. M., Kao K. C. (1998). J. Appl. Phys..

[cit39] Liu Q., Richard C., Capsal J. F. (2017). Eur. Polym. J..

[cit40] Klein R. J., Runt J., Zhang Q. M. (2003). Macromolecules.

[cit41] Taniguchi N., Fukao K., Sotta P., Long D. R. (2015). Phys. Rev. E: Stat., Nonlinear, Soft Matter Phys..

[cit42] Pelrine R. and Kornbluh R., in Electromechanically Act. Polym. A Concise Ref., ed. F. Carpi, Springer International Publishing, Cham, 2016, pp. 671–686

[cit43] Le M. Q., Capsal J. F., Galineau J., Ganet F., Yin X., Yang M. D., Chateaux J. F., Renaud L., Malhaire C., Cottinet P. J., Liang R. (2015). Sci. Rep..

[cit44] Yin X., Lallart M., Cottinet P. J., Guyomar D., Capsal J. F. (2016). Appl. Phys. Lett..

[cit45] Capsal J.-F., Galineau J., Le M.-Q., Domingues Dos Santos F., Cottinet P.-J. (2015). J. Polym. Sci., Part B: Polym. Phys..

[cit46] Chiu F.-C., Lee C.-Y., Pan T.-M. (2009). J. Appl. Phys..

[cit47] Lösche A. (1972). Krist. Tech..

[cit48] Chiu F.-C., Shih W.-C., Feng J.-J. (2012). J. Appl. Phys..

[cit49] Ho J., Jow T. R. (2012). IEEE Trans. Dielectr. Electr. Insul..

[cit50] Lawson W. G. (1965). Br. J. Appl. Phys..

[cit51] McCubbin W. L. (1962). Trans. Faraday Soc..

[cit52] Miyamoto T., Shibayama K. (1973). J. Appl. Phys..

[cit53] Mackey M., Schuele D. E., Zhu L., Baer E. (2012). J. Appl. Phys..

[cit54] Zhou Z., Carr J., Mackey M., Yin K., Schuele D., Zhu L., Baer E. (2013). J. Polym. Sci., Part B: Polym. Phys..

[cit55] Mackey M., Schuele D. E., Zhu L., Flandin L., Wolak M. A., Shirk J. S., Hiltner A., Baer E. (2012). Macromolecules.

[cit56] Kao K. C. , in Dielectr. Phenom. Solids, ed. K. C. Kao, Academic Press, San Diego, 2004, pp. 41–114

[cit57] Yang L., Ho J., Allahyarov E., Mu R., Zhu L. (2015). ACS Appl. Mater. Interfaces.

[cit58] Chen X., Tseng J.-K., Treufeld I., Mackey M., Schuele D., Li R., Fukuto M., Baer E., Zhu L. (2017). J. Mater. Chem. C.

[cit59] Tsutsumi N., Okumachi K., Kinashi K., Sakai W. (2017). Sci. Rep..

[cit60] Smith R. C., Liang C., Landry M., Nelson J. K., Schadler L. S. (2008). IEEE Trans. Dielectr. Electr. Insul..

[cit61] Roy M., Nelson J. K., MacCrone R. K., Schadler L. S., Reed C. W., Keefe R. (2005). IEEE Trans. Dielectr. Electr. Insul..

[cit62] Capsal J.-F., Lallart M., Galineau J., Cottinet P.-J., Sebald G., Guyomar D. (2012). J. Phys. D: Appl. Phys..

[cit63] Lallart M., Capsal J. F., Sebald G., Cottinet P. J., Guyomar D. (2014). Sens. Actuators, B.

[cit64] Pelrine R. E., Kornbluh R. D., Joseph J. P. (1998). Sens. Actuators, A.

[cit65] Zhang Q. M., Bharti V., Zhao X. (1998). Sci. 80.

[cit66] Zhang Q. M. , Cheng Z.-Y., Bharti V., Xu T.-B., Xu H., Mai T. X., and Gross S. J., in Proc. SPIE, 2000

[cit67] Zhang Q. M. , Bharti V., Cheng Z.-Y., Xu T.-B., Wang S., Romotowski T. S., Tito F. A., and Ting R. Y., in Proc. SPIE, 1999

[cit68] Li F., Jin L., Xu Z., Zhang S. (2014). Appl. Phys. Rev..

